# Association Between Preoperative Penile Circumference and Urinary Function After Robot‐Assisted Radical Prostatectomy

**DOI:** 10.1111/iju.70179

**Published:** 2025-07-18

**Authors:** Yuki Kohada, Hiroyuki Kitano, Shinsaku Tasaka, Yuto Ono, Ryo Tasaka, Shunsuke Miyamoto, Tomoya Hatayama, Hiroyuki Shikuma, Kenshiro Takemoto, Miki Naito, Kohei Kobatake, Yohei Sekino, Keisuke Goto, Akihiro Goriki, Keisuke Hieda, Nobuyuki Hinata

**Affiliations:** ^1^ Department of Urology Hiroshima University Graduate School of Biomedical Sciences Hiroshima Japan

**Keywords:** prostatectomy, prostatic neoplasms, quality of life, robot‐assisted surgery, urinary incontinence

## Abstract

**Objectives:**

This study investigated whether preoperative penile length, penile circumference, and testis size are associated with urinary function and sexual function after robot‐assisted radical prostatectomy (RARP).

**Methods:**

We retrospectively analyzed 197 Japanese patients who underwent RARP. Patients were categorized based on the median preoperative penile length, penile circumference, and testis size. Urinary function and sexual function were assessed based on the daily pad usage and the results of the Expanded Prostate Cancer Index Composite (EPIC), International Prostate Symptom Score (IPSS), and International Index of Erectile Function‐5 (IIEF‐5) questionnaires preoperatively and 1, 3, 6, and 12 months postoperatively.

**Results:**

Preoperative penile circumference was significantly associated with postoperative urinary outcomes. The thick penis group (larger than the median preoperative penile circumference) used fewer daily pads and had better urinary function scores (EPIC and IPSS) compared to the thin penis group (median or smaller preoperative penile circumference) postoperatively. In contrast, preoperative penile length exhibited no significant relationship with postoperative urinary function. Postoperative sexual function scores (EPIC and IIEF‐5) showed trends that favored the long (longer than the median preoperative penile length) and thick penis groups than the short (median or shorter preoperative penile length) and thin penis groups, but significant differences were limited to specific time points. Preoperative testis size exhibited no significant relationship with urinary and sexual outcomes.

**Conclusions:**

The preoperative penile circumference was associated with urinary function after RARP, highlighting its potential as a practical clinical marker. However, the association between preoperative penile size and sexual function was minimal.

**Abbreviations**
BMI: body mass index
ED: erectile dysfunction
EPIC: Expanded Prostate Cancer Index Composite
IIEF‐5: International Index of Erectile Function‐5
IPSS: International Prostate Symptom Score
MUL: membranous urethral length
PC: prostate cancer
QOL: quality of life
RARP: robot‐assisted radical prostatectomy

## 1 Introduction

Robot‐assisted radical prostatectomy (RARP) has become the gold standard of treatment for localized prostate cancer (PC) and is performed worldwide [1]. RARP provides satisfactory oncological and functional outcomes; however, postoperative urinary disorders and sexual dysfunction are significant challenges that adversely affect the quality of life (QOL) and treatment satisfaction of patients [2, 3].

The incidence and severity of these complications are influenced by patient characteristics. Therefore, subjective assessments by patients and objective assessments by physicians are necessary to accurately determine patient characteristics associated with these complications. Previous studies have identified preoperative lower urinary tract symptoms and erectile dysfunction (ED) as predictors of postoperative outcomes, and these factors were primarily determined based on subjective patient‐reported assessments [4, 5]. Recent technological advances, such as the use of preoperative magnetic resonance imaging to assess the membranous urethral length (MUL) and neurovascular bundle, have resulted in objective evaluation methods; however, their complexity limits their routine clinical application [6]. Therefore, novel, simple, and reproducible predictive factors that can be evaluated objectively need to be determined.

We focused on the preoperative external genitalia size as a novel objective indicator of functional outcomes after RARP. External genitalia can be easily measured. Basically, congenital factors such as fetal androgen action largely determine the external genitalia size [7]. However, it has been reported that penile size and testis size decrease with aging because of the fibrosis of the tunica albuginea [8, 9]. Studies have shown that patients with ED tend to have a smaller penis [10]. Although a potential relationship between the external genitalia size, age, and sexual function has been reported, no study has addressed an association with functional outcomes after RARP.

This study aimed to determine whether the preoperative external genitalia size is associated with urinary disorders and sexual dysfunction after RARP. We compared serial changes of the postoperative urinary and sexual functions by dividing them into groups based on the preoperative penile length, penile circumference, and testis size. Furthermore, we evaluated the relationship between clinicopathological factors and these parameters of patients who underwent RARP to elucidate the underlying pathophysiological conditions associated with the postoperative urinary and sexual functions.

## 2 Methods

### 2.1 Patients and Study Design

This retrospective study included Japanese patients who underwent RARP at Hiroshima University Hospital between May 2010 and September 2023. The study was approved by the Hiroshima University Ethics Committee (E2022‐0003). All surgeries were performed using the transperitoneal approach, as previously described [11]. Procedures were conducted by urologists certified as proctors under the Robotic Surgery Proctor Certification System of the Japanese Society of Endourology and Robotics, ensuring sufficient expertise in robotic‐assisted surgery. Nerve‐sparing procedures were performed based on grading, as described by Tewari et al. [12] Surgeries with nerve‐sparing of intra‐fascial or inter‐fascial dissection on at least one side were defined as nerve‐sparing procedures in this study, with consideration to cancer risk and patient request. Throughout the study period, external genital size was routinely measured at the time of prostate biopsy in our institution. Patients who were diagnosed with PC on biopsy and subsequently underwent RARP were included in the study if external genital size measurements were available. Patients were included if preoperative data and the results of at least one postoperative follow‐up assessment (at 1, 3, 6, or 12 months) such as the Expanded Prostate Cancer Index Composite (EPIC), International Prostate Symptom Score (IPSS), and International Index of Erectile Function‐5 (IIEF‐5) questionnaires were available [13, 14, 15]. Patients who underwent salvage therapy because of biochemical recurrence within 12 months or neoadjuvant hormonal therapy were excluded.

### 2.2 Exposure Measures

The penile length, penile circumference, and testis size were measured preoperatively. Penile length was defined as the length from the suprapubic skin to the distal glans along the dorsal side of the penis, and penile circumference was measured at the mid‐shaft [16]. The testis size was measured using a punched‐out orchidometer [17]. The orchidometer consisted of 16 punched‐out elliptical rings with graded volumes of 1–30 mL (1–6, 8, 10, 12, 14, 16, 18, 20, 22, 24, and 26 mL). A ring was placed over the stretched scrotal skin and up to the mid‐portion of the testis, away from the epididymis. If the ring was easily placed, then placement of a smaller ring was attempted, and the testis was compressed to fit the ring. All measurements were performed at room temperature (24°C) with the patient in the lithotomy position at the time of prostate biopsy and in a flaccid state with the penis extended to maximum capacity at a 90° angle to the body. Figure S1 illustrates the measurement techniques for clarity.

Penile length, penile circumference, and testis size were categorized as long or short, thick or thin, and large or small, respectively, based on whether the measurements were larger than or smaller than the median.

### 2.3 Assessment for Urinary Symptoms and Sexual Functions

All patients filled out the EPIC, IPSS, and IIEF‐5 questionnaires preoperatively and 1, 3, 6, and 12 months postoperatively. The EPIC questionnaire is a valid and reliable tool used to analyze the patient function and inconveniences experienced after PC treatment [13]. The EPIC urinary and sexual subdomains (urinary function and bother, incontinence, irritation/obstruction, sexual function and bother) were analyzed. Question 5 of the EPIC questionnaire inquired about the number of pads daily used. Urinary continence was defined as using no pad, and urinary incontinence was defined as using ≥ 1 pad/day, including a safety pad. The IPSS comprises seven questions that assess voiding symptoms (incomplete emptying, intermittency, weak stream, and straining to void) and storage symptoms (frequency, urgency, and nocturia), and one question that assesses the QOL (IPSS‐QOL) [14]. The IIEF‐5 is an abridged 5‐item version of the IIEF‐15 that is used to evaluate the presence and severity of ED [15]. All these questionnaires were translated into Japanese.

### 2.4 Data Collection

Relevant clinicopathological data, including age, body mass index (BMI), hypertension, diabetes mellitus, cardiovascular disease, medication status for benign prostate hyperplasia, preoperative serum total testosterone and prostate‐specific antigen levels, prostate volume (measured using transabdominal ultrasonography), nerve‐sparing procedure, American Society of Anesthesiologists physical status, penile rehabilitation, pathological grade of the prostatic biopsy specimen (classified using the International Society of Urological Pathology grading) [18], and pathological *T*‐stage were retrospectively collected. Penile rehabilitation with weekly PDE5i was provided upon patient request.

### 2.5 Statistical Analysis

Continuous variables were reported as medians with interquartile ranges (IQRs) and compared using the Wilcoxon rank‐sum test. Categorical variables were analyzed using Pearson's chi‐square test. To assess inter‐examiner variability in the measurement of external genital size, a one‐way analysis of variance (ANOVA) was performed for each parameter. Pearson correlation analyses were performed to evaluate the linear associations among external genital measurements. Univariate and multivariate logistic regression analyses were performed to identify predictors of urinary continence and other functional scores at 12 months using genital and clinical parameters. Statistical significance was set at *p* < 0.05, and analyses were conducted using JMP Pro 18.0.0 (SAS Institute Inc.).

## 3 Results

### 3.1 Patient Characteristics

A total of 197 patients were included in this study (Table 1). The median preoperative penile length and circumference were both 8.0 cm (IQR, 7.0–9.0 cm and 7.5–9.0 cm, respectively). The median preoperative testis size was 20 mL (IQR, 16–24 mL for the left testis; IQR, 16–22 mL for the right testis), with no asymmetry. The median total IPSS was 7 (IQR, 4–13), indicating mild lower urinary tract symptoms. In contrast, the median total IIEF‐5 score was 5 (IQR, 2–16), suggesting that most patients had severe ED. A total of 41 physicians were involved in the measurements, with a mean of 4.8 cases per physician. The corresponding means and standard deviations are shown in Table S1. The ANOVA results demonstrated significant variability among physicians for testis size (*F* = 3.02, *p* = 0.001) and penile length (*F* = 4.03, *p* = 0.001) but not for penile circumference (*F* = 2.10, *p* = 0.061). Pearson correlation analysis revealed a weak but significant correlation between penile circumference and left testis size (*r* = 0.263, *p* < 0.001), while no significant correlations were observed between the other measurements (Figure S2).

**TABLE 1:** Baseline characteristics of all patients.

Variables	Overall
	*n* = 197
Age (years): median (IQR)	69 (65–73)
BMI (kg/m^2^): median (IQR)	22.9 (21.7–25.2)
HT: *n* (%)	79 (40.1)
DM: *n* (%)	35 (17.8)
CVD: *n* (%)	33 (16.8)
Medication for BPH: *n* (%)	54 (27.4)
Total testosterone: median (IQR)	4.5 (3.5–5.6)
Prostate volume, (mL): median (IQR)	29 (22–38)
Initial PSA (ng/mL): median (IQR)	7.3 (5.1–10.7)
Pathological grade: *n* (%)	
1, 2	74 (37.6)
3, 4, 5	123 (62.4)
Pathological *T* stage: *n* (%)	
T1c, T2a, T2b, T2c	170 (86.3)
T3a, T3b	27 (13.7)
Nerve sparing: *n* (%)	56 (28.4)
ASA‐PS: *n* (%)	
1	6 (3.0)
2	191 (97.0)
3	0 (0.0)
Penile rehabilitation	9 (4.6)
Penile length (cm): median (IQR)	8.0 (7.0–9.0)
Penile circumference (cm): median (IQR)	8.0 (7.5–9.0)
Testis size (mL)	
Left: median (IQR)	20 (16–24)
Right: median (IQR)	20 (16–22)
EPIC score subdomain	
Urinary function: median (IQR)	100 (95.0–100)
Urinary bother: median (IQR)	89.3 (82.1–100)
Urinary incontinence: median (IQR)	100 (93.8–100)
Urinary irritation/obstruction: median (IQR)	92.9 (85.7–96.4)
Sexual function: median (IQR)	19.9 (5.6–45.4)
Sexual bother: median (IQR)	93.8 (75.0–100)
Total IPSS: median (IQR)	7 (4–13)
IPSS‐voiding: median (IQR)	3 (1–8)
IPSS‐storage: median (IQR)	3 (2–6)
IPSS‐QOL: median (IQR)	3 (1–4)
Total IIEF‐5 score: median (IQR)	5 (2–16)

Abbreviations: ASA‐PS, American Society of Anesthesiologists physical status; BMI, body mass index; BPH, Benign prostatic hyperplasia; CVD, cardiovascular disease; DM, diabetes mellitus; EPIC, expanded prostate cancer Index composite; HT, hypertension; IIEF‐5, International Index of Erectile Function‐5; IPSS, international prostate symptom score; IQR, interquartile range; PSA, prostate‐specific antigen; QOL, quality of life.

### 3.2 Groupwise Comparison of Serial Changes in the Urinary Function and Sexual Function After RARP Based on the Preoperative Penile Length, Penile Circumference, and Testis Size

Patients were divided into groups based on the median preoperative penile length (long penis group, > 8.0 cm [*n* = 94]; short penis group, ≤ 8.0 cm [*n* = 103]), preoperative penile circumference (thick penis group, > 8.0 cm [*n* = 92]; thin penis group, ≤ 8.0 cm [*n* = 105]), and preoperative left testis size (large testis group, > 20 mL [*n* = 93]; small testis group, ≤ 20 mL [*n* = 104]). The response rates for the questionnaires were 100% (*n* = 197), 85.3% (*n* = 168), 87.8% (*n* = 173), 86.8% (*n* = 171), and 85.3% (*n* = 168) preoperatively and 1, 3, 6, and 12 months postoperatively, respectively.

**FIGURE 1:**
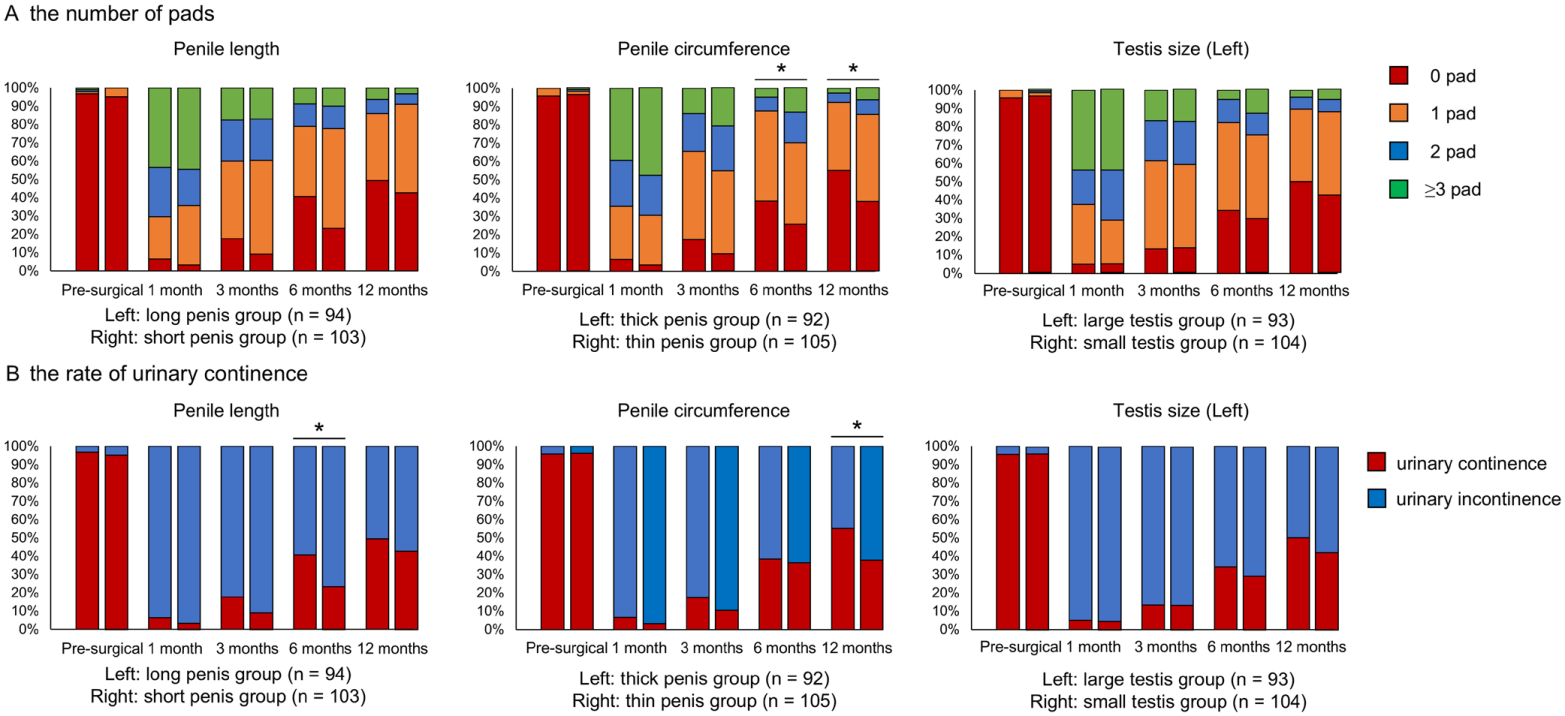
Comparisons of longitudinal changes in the number of pads used daily (A) and the rate of urinary continence (B) after robot‐assisted radical prostatectomy (RARP) among the long penis (> 8.0 cm, *n* = 94) and short penis (≤ 8.0 cm, *n* = 103) groups, thick penis (> 8.0 cm, *n* = 92) and thin penis (≤ 8.0 cm, *n* = 105) groups, and large testis (> 20 mL, *n* = 93) and small testis (≤ 20 mL, *n* = 104) groups. *p < 0.05.

**FIGURE 2:**
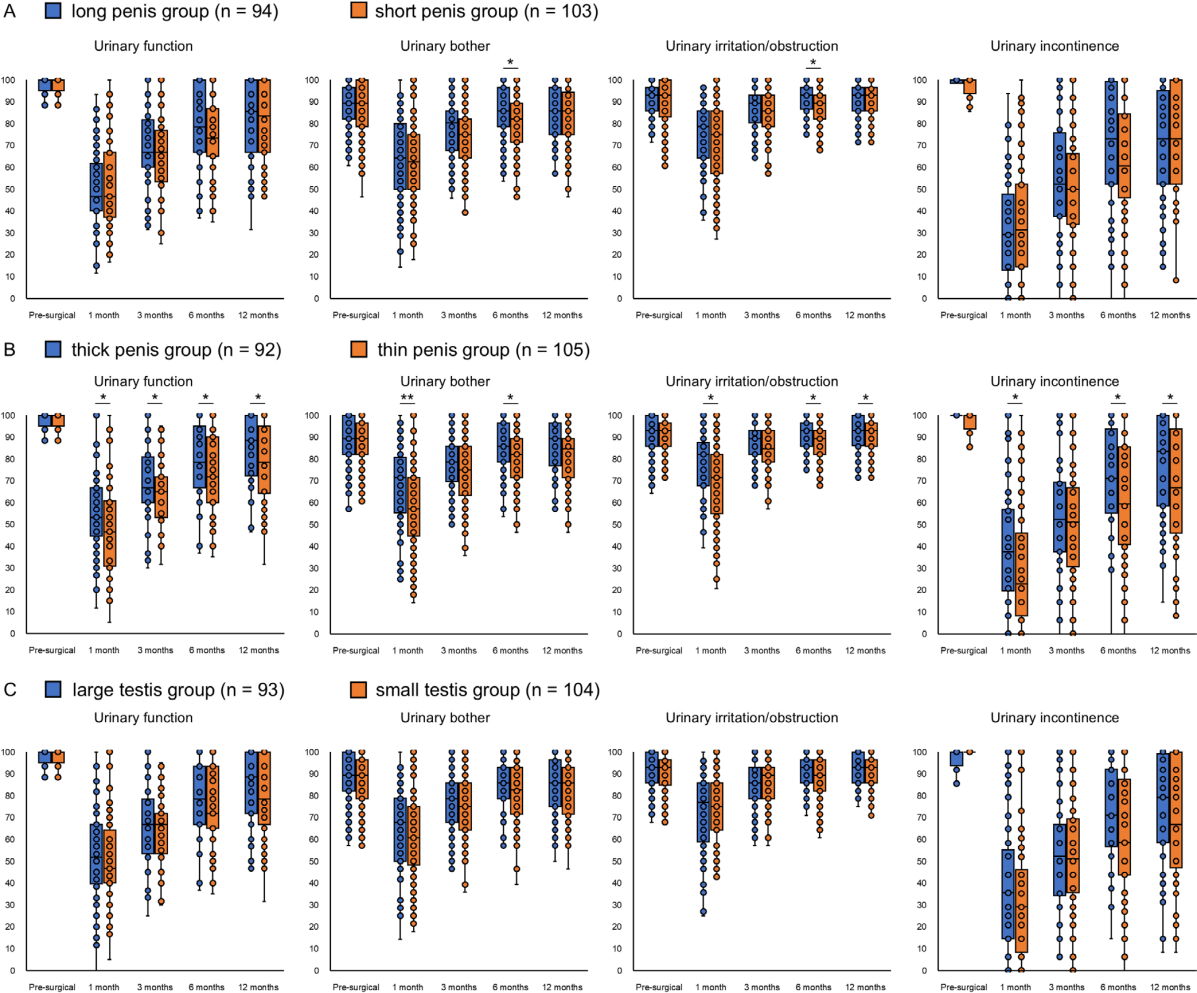
Comparisons of longitudinal changes in the Expanded Prostate Cancer Index Composite (EPIC) urinary subdomain scores among the long penis and short penis groups, thick penis and thin penis groups, and large testis and small testis groups. (A) Changes in the long penis (> 8.0 cm, *n* = 94) and short penis (≤ 8.0 cm, *n* = 103) groups. (B) Changes in the thick penis (> 8.0 cm, *n* = 92) and thin penis (≤ 8.0 cm, *n* = 105) groups. (C) Changes in the large testis (> 20 mL, *n* = 93) and small testis (≤ 20 mL, *n* = 104) groups. *p < 0.05 and **p < 0.001.

**FIGURE 3:**
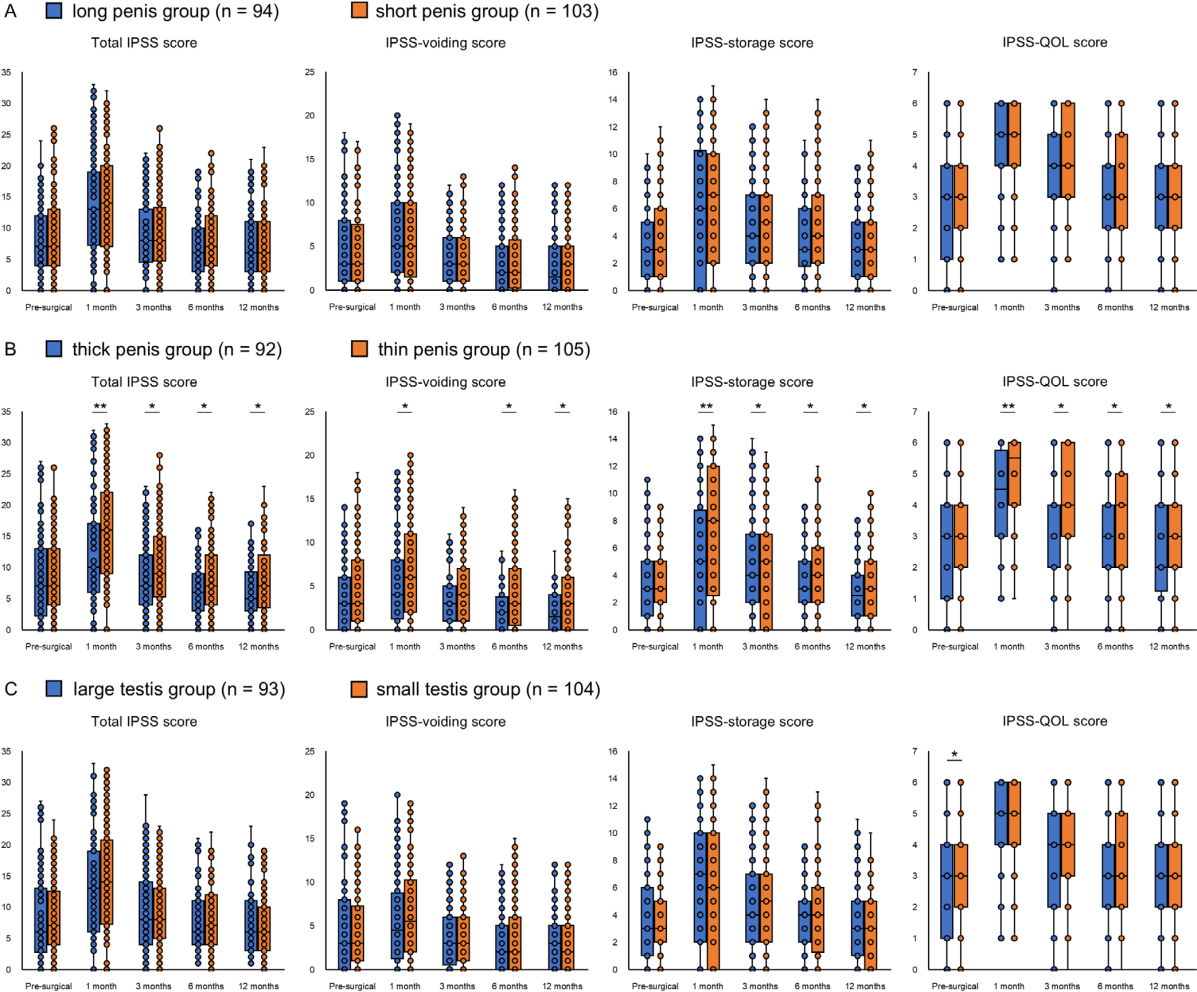
Comparisons of longitudinal changes in the total International Prostate Symptom Score (IPSS), IPSS‐voiding/storage, and IPSS‐quality of life (QOL) among the long penis and short penis groups, thick penis and thin penis groups, and large testis and small testis groups. (A) Changes in the long penis (> 8.0 cm, *n* = 94) and short penis (≤ 8.0 cm, *n* = 103) groups. (B) Changes in the thick penis (> 8.0 cm, *n* = 92) and thin penis (≤ 8.0 cm, *n* = 105) groups. (C) Changes in the large testis (> 20 mL, *n* = 93) and small testis (≤ 20 mL, *n* = 104) groups. *p < 0.05 and **p < 0.001.

First, we compared serial changes in urinary function between groups. Compared to the thin penis group, the thick penis group used significantly fewer daily pads at 6 and 12 months postoperatively (Figure 1). Regarding urinary continence, the long penis group showed a significantly higher continence rate than the short penis group at 6 months postoperatively, while the thick penis group showed a significantly higher rate than the thin penis group at 12 months. Additionally, compared to the thin penis group, the thick penis group had consistently better EPIC urinary subdomain scores, total IPSS, and IPSS voiding/storage symptom subdomain postoperatively (Figures 2, 3). Interestingly, the IPSS‐QOL, which evaluates the QOL related to urinary function, of the thick penis group was also significantly better than that of the thin penis group postoperatively. In contrast, no significant differences were observed between the long and short penis groups or between the large and small testis groups. Univariate and multivariate logistic regression analyses revealed that penile circumference (> 8.0 cm) independently predicted continence and total IPSS at 12 months (Table 2 and Tables S2 and S3). No significant associations were found for external genital parameters and EPIC or IPSS‐QOL scores.

**TABLE 2:** Univariate and multivariate logistic regression analyses for predictors of urinary continence.

Clinicopathological factors	Univariate analysis	Multivariate analysis
Age (≥ 70 years vs. < 70 years)		
OR (95% CI)	1.19 (0.65–2.19)	1.36 (0.71–2.60)
*p*	0.578	0.349
Medication for BPH		
OR (95% CI)	0.75 (0.38–1.47)	0.72 (0.34–1.50)
*p*	0.401	0.377
Prostate volume (≥ 30 mL vs. < 30 mL)		
OR (95% CI)	1.30 (0.71–2.40)	1.39 (0.72–2.67)
*p*	0.396	0.324
Nerve sparing		
OR (95% CI)	1.69 (0.87–3.30)	1.72 (0.86–3.42)
*p*	0.120	0.123
Penile length (≥ 8.0 cm vs. < 8.0 cm)		
OR (95% CI)	1.31 (0.71–2.41)	1.06 (0.55–2.03)
*p*	0.386	0.857
Penile circumference (≥ 8.0 cm vs. < 8.0 cm)		
OR (95% CI)	2.02 (1.10–3.78)	2.00 (1.04–3.83)
*p*	0.024*	0.038*
Testis size (≥ 20 mL vs. < 20 mL)		
OR (95% CI)	1.37 (0.74–2.53)	1.16 (0.60–2.24)
*p*	0.313	0.655

Abbreviations: BPH, benign prostate hyperplasia; CI, confidence interval; OR, odds ratio.

* *p* < 0.05.

**FIGURE 4:**
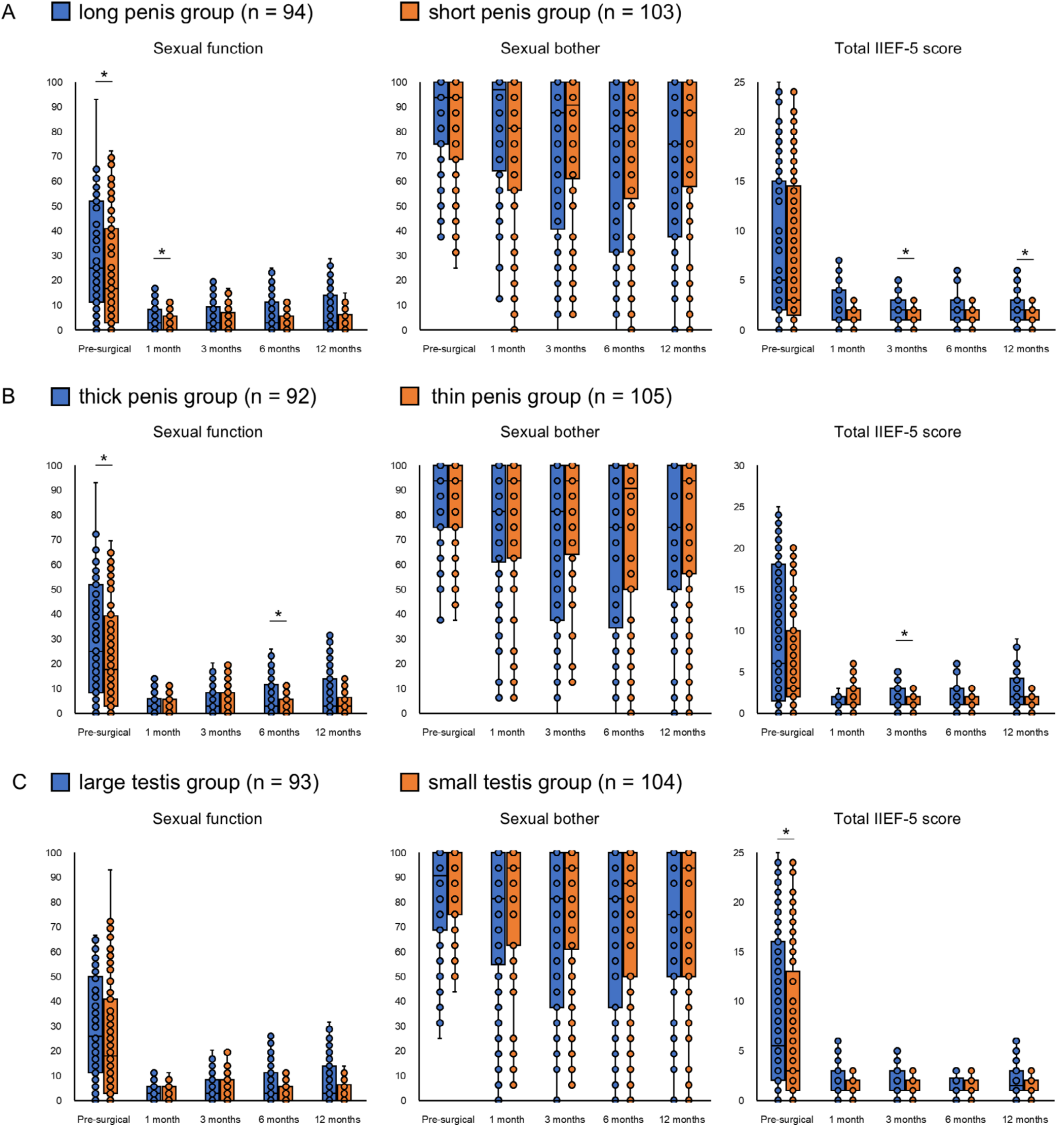
Comparisons of longitudinal changes in the Expanded Prostate Cancer Index Composite (EPIC) urinary subdomain and total International Index of Erectile Function‐5 (IIEF‐5) scores among the long penis and short penis groups, thick penis and thin penis groups, and large testis and small testis groups. (A) Changes in the long penis (> 8.0 cm, *n* = 94) and short penis (≤ 8.0 cm, *n* = 103) groups. (B) Changes in the thick penis (> 8.0 cm, *n* = 92) and thin penis (≤ 8.0 cm, *n* = 105) groups. (C) Changes in the large testis (> 20 mL, *n* = 93) and small testis (≤ 20 mL, *n* = 104) groups. *p < 0.05.

A similar comparison of serial changes in sexual function was performed (Figure 4). Preoperative sexual function scores of the long penis and thick penis groups were higher than those of the short penis and thin penis groups. Postoperative sexual function and IIEF‐5 scores showed trends that favored the long and thick penis groups, although significant differences were limited to specific time points. No significant differences in sexual bother scores based on penile length or circumference were observed. Regarding testis size, the only significant difference observed was the better preoperative IIEF‐5 score of the large testis group.

### 3.3 Comparison of Clinicopathological Characteristics Based on Penile Length, Penile Circumference, and Testis Size

Clinicopathological factors (Table 3) revealed that the thick penis group had a significantly higher BMI and lower cardiovascular disease rate than those of the thin penis group. No significant differences in other factors related to penile length or testicular size were observed.

**TABLE 3:** Comparison of clinicopathological characteristics based on penile length, penile circumference, and testis size.

	Penile circumference			Penile length			Testis size		*p*
	Thick penis group (> 8.0 cm) *n* = 92	Thin penis group (≤ 8.0 cm) *n* = 105	*p*	Long penis group (> 8.0 cm) *n* = 94	Short penis group (≤ 8.0 cm) *n* = 103	*p*	Large testis group (> 20 mL) *n* = 93	Small testis group (≤ 20 mL) *n* = 104	
Age: median (IQR)	69 (63–72)	70 (66–72)	0.110	69 (65–73)	69 (65–73)	0.752	69 (65–73)	69 (65–73)	0.699
BMI: median (IQR)	23.5 (21.9–25.5)	22.4 (21.4–23.9)	0.006*	22.8 (21.6–24.0)	22.9 (21.7–25.5)	0.259	22.8 (21.6–24.0)	22.9 (21.7–25.5)	0.690
HT: *n* (%)	33 (35.9)	46 (43.8)	0.256	32 (34.0)	47 (45.6)	0.097	36 (38.7)	43 (41.4)	0.706
DM: *n* (%)	15 (16.3)	20 (19.1)	0.615	15 (16.0)	20 (19.4)	0.525	13 (14.0)	22 (21.2)	0.186
CVD: *n* (%)	9 (9.8)	24 (22.9)	0.013*	11 (11.7)	22 (21.4)	0.067	16 (17.2)	17 (16.4)	0.872
Medication for BPH: *n* (%)	22 (23.4)	32 (31.1)	0.227	23 (25.0)	31 (29.5)	0.477	18 (19.4)	36 (34.6)	0.016*
Total Testosterone: median (IQR)	4.4 (3.3–5.6)	4.6 (3.8–5.5)	0.323	4.5 (3.7–5.5)	4.5 (3.3–5.6)	0.699	4.5 (3.7–5.5)	4.5 (3.3–5.7)	0.641
Prostate volume: median (IQR)	27 (22–39)	30 (22–37)	0.899	30 (24–39)	27 (21–37)	0.111	28 (22–37)	30 (23–38)	0.185
Initial PSA (ng/mL): median (IQR)	7.1 (5.0–11.0)	7.7 (5.1–10.4)	0.668	7.9 (5.1–11.6)	7.0 (5.0–11.6)	0.417	7.6 (4.9–11.2)	7.0 (5.2–10.4)	0.866
Nerve sparing: *n* (%)	27 (28.7)	29 (28.2)	0.930	27 (29.4)	29 (27.6)	0.789	30 (32.3)	26 (25.0)	0.260
ASA‐PS: *n* (%)			0.059			0.066			0.330
1	5 (5.4)	1 (1.0)		5 (5.3)	1 (1.0)		4 (4.3)	2 (1.9)	
2	87 (94.6)	104 (99.0)		89 (94.7)	102 (99.0)		89 (95.7)	102 (98.1)	
3	0 (0.0)	0 (0.0)		0 (0.0)	0 (0.0)		0 (0.0)	0 (0.0)	
Penile rehabilitation	7 (7.6)	2 (1.9)	0.051	5 (5.3)	4 (3.9)	0.630	4 (4.3)	5 (4.8)	0.865
Pathological grade ≥ 3: *n* (%)	56 (60.9)	67 (63.8)	0.671	59 (62.8)	64 (62.1)	0.927	58 (62.4)	65 (62.5)	0.985
Pathological *T* stage ≥ 3: *n* (%)	12 (13.0)	15 (14.3)	0.800	12 (12.8)	15 (14.6)	0.714	11 (11.8)	16 (15.4)	0.467

Abbreviations: ASA‐PS, American Society of Anesthesiologists physical status; BMI, body mass index; BPH, benign prostate hyperplasia; CVD, cardiovascular disease; DM, diabetes mellitus; HT, hypertension; IQR, interquartile range; PSA, prostate‐specific antigen.

* *p* < 0.05.

## 4 Discussion

We investigated the relationship between preoperative external genitalia size and functional outcomes after RARP. To the best of our knowledge, this is the first study to identify an association between preoperative penile circumference and postoperative urinary function. On the other hand, the relationship between the preoperative penile circumference and postoperative sexual function was limited. Because it can be measured without the need for imaging modalities such as MRI, preoperative penile circumference may serve as a practical predictor of urinary disorders after RARP.

Preoperative penile circumference was the only external genitalia parameter associated with urinary disorders after RARP. This finding may be explained by fibrosis of the corpora cavernosa, induced by pelvic atherosclerosis, with the penile circumference potentially reflecting the degree of fibrosis. Anatomically, the penis consists of the corpora cavernosa and the urethra; the corpora cavernosa size largely determines penile circumference [19]. After prostatectomy, fibrosis of the corpora cavernosa is induced, resulting in a reduced penile size [20]. Such fibrosis can also occur because of hypoxia and reduced nitric oxide synthase‐containing nerve fibers caused by pelvic atherosclerosis [21]. Consistent with this, cardiovascular disease, which is a condition linked to atherosclerosis, was more prevalent in the thin penis group. Preoperative ED, which is often associated with atherosclerosis [22], is a predictor of postoperative urinary incontinence [5]. Because preoperative penile circumference and sexual function were correlated in this study, penile circumference may serve as a surrogate marker of pelvic atherosclerosis and its effects (including ED) potentially contributing to postoperative urinary dysfunction after RARP.

Because penile circumference may reflect pelvic atherosclerosis and its effects, we hypothesized that patients in the thin penis group would experience worse postoperative sexual function compared to those in the thick penis group. However, associations between preoperative penile circumference and postoperative EPIC sexual function or IIEF‐5 scores were minimal. This may be attributable to the low baseline sexual function of Japanese patients, who generally exhibit poorer recovery of sexual function after RARP compared to that of patients in Western populations [23]. In our cohort, many patients had severe ED preoperatively, as determined based on the IIEF‐5 score. The predictive value of the penile size for postoperative sexual function may be limited to patients with high preoperative sexual function, nerve‐sparing surgery, or postoperative penile rehabilitation.

Preoperative penile length and testis size were not associated with functional outcomes after RARP. The absence of a relationship between preoperative penile length and postoperative urinary disorders may reflect measurement variability. In this study, penile length was assessed by stretching from the pubic skin junction to the glans tip, which is a method prone to inconsistency due to examiner‐applied traction, prepubic fat, and phimosis [24]. These factors create challenges obtaining reproducible measurements. In contrast, penile circumference was less affected by these variables, resulting in more consistent and reliable measurements. This methodological advantage may explain why penile circumference was significantly associated with urinary outcomes, whereas penile length was not. The ANOVA results demonstrated that penile circumference measurements were less variable across different physicians compared to penile length. Although anatomical differences may underlie their distinct relationships with outcomes, further research is needed to clarify the mechanisms.

The findings that preoperative penile circumference is associated with urinary disorders after RARP have valuable clinical implications for physicians. Preoperative penile circumference may serve as an independent risk factor for urinary disorders that is distinct from non‐modifiable factors such as age [25], prostate volume [26], and MUL [27], because it may serve as a surrogate marker for pelvic atherosclerosis. Unlike MUL, the penile circumference is simple to measure, thus making it a practical indicator in clinical settings. Because consistently lower postoperative IPSS‐QOL scores were observed in the thin penis group, these patients may benefit from proactive preoperative interventions, such as pelvic floor muscle training, similar to those for elderly patients or individuals with a short MUL [27].

Interestingly, the thick penis group had a high BMI, which contrasted with the findings of previous studies that associated high BMI values with urinary disorders [28, 29]. This discrepancy may be attributable to the relatively lower BMI of our cohort; in this study, higher BMI values likely reflected a better nutritional status and more muscle mass. Muscle mass is known to influence urinary incontinence after radical prostatectomy [30], and interventions that target muscle preservation may be especially beneficial for the thin penis group. Further physiological studies are required to validate this hypothesis.

This study has several limitations. First, this was a single‐center study with a small sample size. Not all patients undergoing RARP at our institution had prostate biopsies performed here, resulting in missing genital size data for many cases and contributing to a smaller sample size. Furthermore, postoperative measurements of external genital size were not available due to the retrospective design. The long observational period and the involvement of multiple physicians may also have introduced variability in the measurements and RARP procedures. The low questionnaire response rates at certain postoperative time points may have influenced the results. Furthermore, our cohort only included Japanese patients. Because Asian patients with PC tend to have lower sexual function before and after radical prostatectomy [23], it is possible that no significant association between external genitalia size and postoperative sexual function was observed in our cohort. Additionally, ethnic differences in external genital size and their potential impact on outcomes warrant further investigation. Despite these limitations, the findings of this study can help physicians make informed treatment decisions for patients with localized PC. Additional prospective multicenter studies are required to verify our results.

In conclusion, preoperative penile circumference was associated with urinary disorders after RARP and may serve as one of the possible associated factors in clinical practice. Preoperative interventions such as pelvic floor muscle training may be particularly beneficial for patients with a smaller preoperative penile circumference.

## Author Contributions

**Yuki Kohada:** conceptualization, data curation, writing – original draft, project administration, visualization, methodology. **Hiroyuki Kitano:** visualization, writing – review and editing. **Shinsaku Tasaka:** data curation. **Yuto Ono:** data curation. **Ryo Tasaka:** data curation. **Shunsuke Miyamoto:** data curation. **Tomoya Hatayama:** data curation. **Hiroyuki Shikuma:** data curation. **Kenshiro Takemoto:** data curation. **Miki Naito:** data curation. **Kohei Kobatake:** data curation. **Yohei Sekino:** data curation. **Keisuke Goto:** data curation. **Akihiro Goriki:** data curation. **Keisuke Hieda:** data curation. **Nobuyuki Hinata:** supervision.

## Ethics Statement

The protocol for this research project has been approved by a suitably constituted Ethics Committee of the institution, and it conforms to the provisions of the Declaration of Helsinki. Committee of Hiroshima University Hospital, Approval No. E2022‐0003. All human subjects provided written informed consent with guarantees of confidentiality.

## Conflicts of Interest

Nobuyuki Hinata is an Editorial Board member of International Journal of Urology and a coauthor of this article. To minimize bias, they were excluded from all editorial decision‐making related to the acceptance of this article for publication.

## Supporting information


**Figure S1.** Illustration of the measurement methods for external genital parameters. (A) Penile length was measured from the suprapubic skin to the distal glans along the dorsal side of the penis in a flaccid state, with the penis extended at a 90° angle to the body. (B) Penile circumference was measured at the mid‐shaft in the same position. (C) Testis size was assessed using a punched‐out orchidometer placed around the mid‐portion of the testis, excluding the epididymis.

**Figure S2.** Scatter plots and linear regression lines illustrating the correlations between external genital parameters. (A) Penile length versus penile circumference (*r* = 0.08, *p* = 0.28); (B) Penile length versus left testis size (*r* = 0.05, *p* = 0.48); (C) Penile circumference vs. left testis size (*r* = 0.26, *p* < 0.001). Pearson correlation analysis was used to evaluate linear associations.

**Table S1.** Mean and standard deviation (SD) for penile length, penile circumference, and left testis size by physicians (≥ 10 measurements) The ANOVA results demonstrated significant variability among physicians for testis size (*F* = 3.02, *p* = 0.001) and penile length (*F* = 4.03, *p* = 0.001) but not for penile circumference (*F* = 2.10, *p* = 0.061).

**Table S2.** Univariate and multivariate logistic regression analyses for predictors of high EPIC urinary subdomains scores. BPH, benign prostate hyperplasia; CI, confidence interval; EPIC, Expanded Prostate Cancer Index Composite; OR, odds ratio. *p < 0.05.

**Table S3.** Univariate and multivariate logistic regression analyses for predictors of high total IPSS and IPSS‐QOL. BPH, benign prostate hyperplasia; CI, confidence interval; IPSS, International Prostate Symptom Score; OR, odds ratio; QOL, quality of life, *p < 0.05.

## Data Availability

Data supporting the findings of this study are available from the corresponding author (HK) upon reasonable request.
