# Supplementary figures and images for: Association Between Preoperative Penile Circumference and Urinary Function After Robot‐Assisted Radical Prostatectomy
Source: Int J Urol. 2025 Jul 18;32(11):1576–86. doi: 10.1111/iju.70179 (PMC12586765; doi:10.1111/iju.70179)

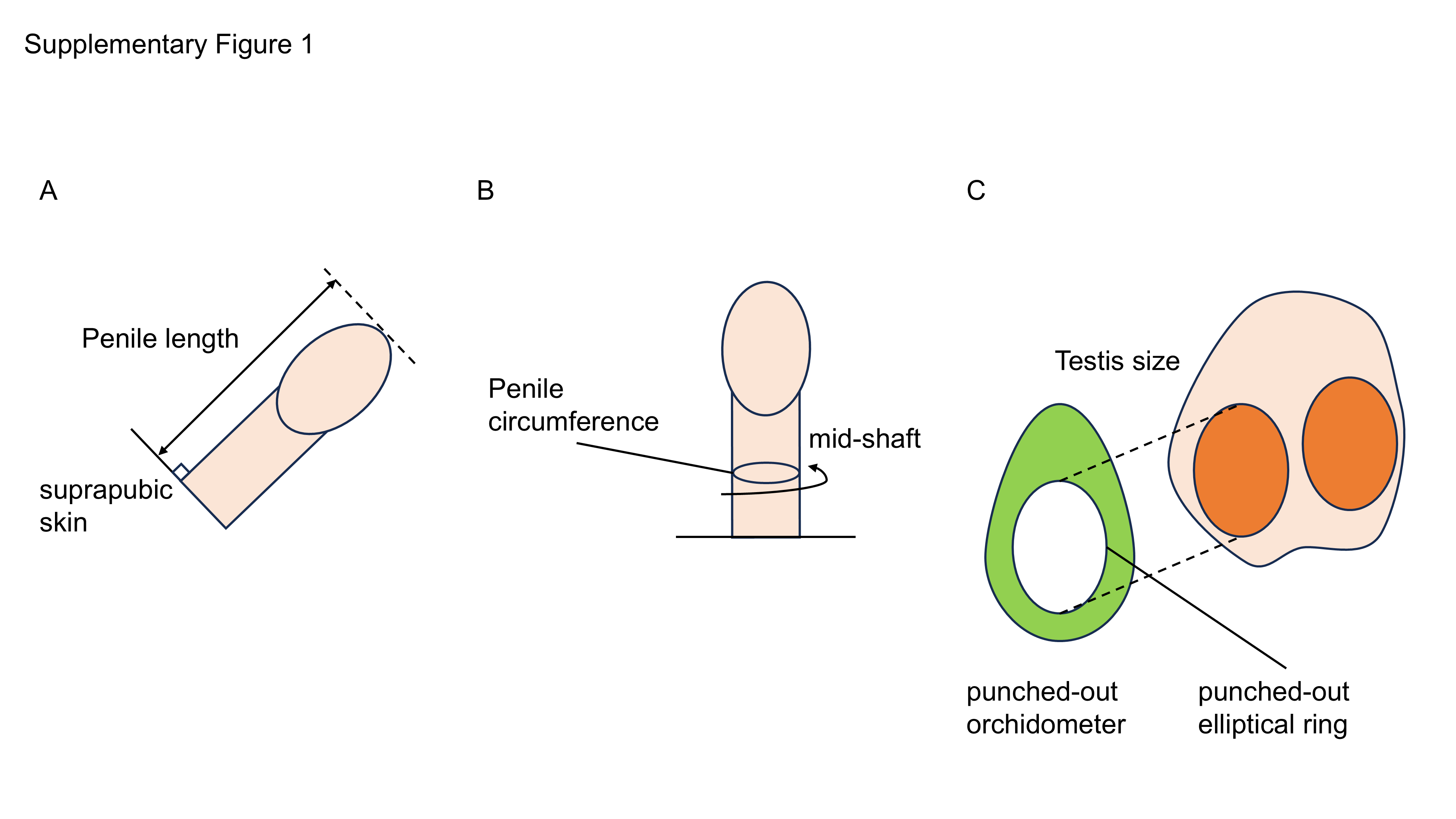

Supplement: Supplementary file 1 — Figure S1. Illustration of the measurement methods for external genital parameters. (A) Penile length was measured from the suprapubic skin to the distal glans along the dorsal side of the penis in a flaccid state, with the penis extended at a 90° angle to the body. (B) Penile circumference was measured at the mid‐shaft in the same position. (C) Testis size was assessed using a punched‐out orchidometer placed around the mid‐portion of the testis, excluding the epididymis. [file IJU-32-1576-s001.tif]

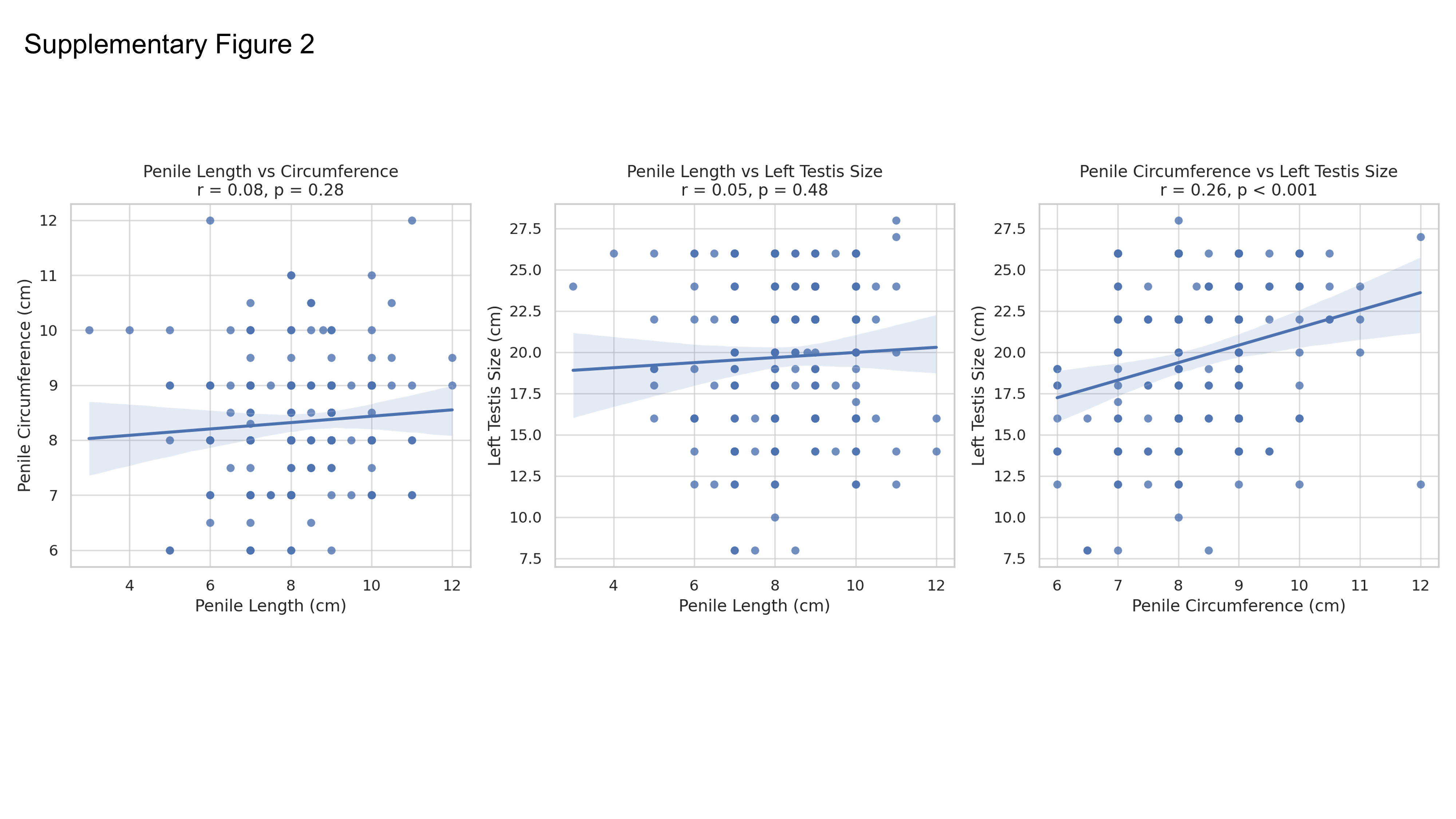

Supplement: Supplementary file 2 — Figure S2. Scatter plots and linear regression lines illustrating the correlations between external genital parameters. (A) Penile length versus penile circumference (r = 0.08, p = 0.28); (B) Penile length versus left testis size (r = 0.05, p = 0.48); (C) Penile circumference vs. left testis size (r = 0.26, p < 0.001). Pearson correlation analysis was used to evaluate linear associations. [file IJU-32-1576-s005.tif]
