# Supplementary material for: Association Between Preoperative Penile Circumference and Urinary Function After Robot‐Assisted Radical Prostatectomy
Source: Int J Urol. 2025 Jul 18;32(11):1576–86. doi: 10.1111/iju.70179 (PMC12586765; doi:10.1111/iju.70179)
Supplement: Supplementary file 3 — Table S1. Mean and standard deviation (SD) for penile length, penile circumference, and left testis size by physicians (≥ 10 measurements) The ANOVA results demonstrated significant variability among physicians for testis size (F = 3.02, p = 0.001) and penile length (F = 4.03, p = 0.001) but not for penile circumference (F = 2.10, p = 0.061). [file IJU-32-1576-s003.docx]

Supplementary Table 1. Mean and standard deviation (SD) for penile length, penile circumference, and left testis size by physicians (≥10 measurements) The ANOVA results demonstrated significant variability among physicians for testis size (F-value = 3.02, p-value = 0.001) and penile length (F-value = 4.03, p-value = 0.001) but not for penile circumference (F-value = 2.10, p-value = 0.061).

| Physicians | Penile length Mean (cm) | Penile length SD (cm) | Penile circumference Mean (cm) | Penile circumference SD (cm) | Left Testis size Mean (mL) | Left Testis size SD (mL) | Measurements |
| --- | --- | --- | --- | --- | --- | --- | --- |
| A | 8.47 | 1.41 | 8.25 | 0.68 | 23.38 | 3.07 | 16 |
| B | 8.37 | 1.08 | 7.89 | 0.74 | 19.05 | 4.84 | 19 |
| C | 7.91 | 2.07 | 7.59 | 2.17 | 19.00 | 3.90 | 11 |
| D | 8.75 | 1.60 | 8.00 | 0.85 | 17.83 | 4.13 | 12 |
| E | 7.35 | 1.25 | 9.15 | 1.20 | 22.20 | 3.71 | 10 |
| F | 8.18 | 0.97 | 8.21 | 0.99 | 20.00 | 4.22 | 14 |
| G | 6.20 | 1.75 | 8.40 | 0.97 | 21.60 | 5.46 | 10 |
