# Supplementary material for: Association Between Preoperative Penile Circumference and Urinary Function After Robot‐Assisted Radical Prostatectomy
Source: Int J Urol. 2025 Jul 18;32(11):1576–86. doi: 10.1111/iju.70179 (PMC12586765; doi:10.1111/iju.70179)
Supplement: Supplementary file 4 — Table S2. Univariate and multivariate logistic regression analyses for predictors of high EPIC urinary subdomains scores. BPH, benign prostate hyperplasia; CI, confidence interval; EPIC, Expanded Prostate Cancer Index Composite; OR, odds ratio. *p < 0.05. [file IJU-32-1576-s002.docx]

Supplementary Table 2

Univariate and multivariate logistic regression analyses for predictors of high EPIC urinary subdomains scores.

| Clinicopathological factors | Urinary function (≥85) | |
| --- | --- | --- |
|  | Univariate analysis | Multivariate analysis |
| Age (≥70 years vs. <70 years) |  |  |
| OR (95%CI) | 1.05 (0.57–1.92) | 1.11 (0.59–2.10) |
| p-value | 0.886 | 0.745 |
| Medication for BPH (Positive vs. negative) |  |  |
| OR (95%CI) | 0.72 (0.37–1.42) | 0.74 (0.36–1.54) |
| p-value | 0.353 | 0.422 |
| Prostate volume (≥30 mL vs. <30 mL) |  |  |
| OR (95%CI) | 1.24 (0.68–2.28) | 1.38 (0.73–2.64) |
| p-value | 0.487 | 0.324 |
| Nerve sparing (Bilateral/Unilateral vs. non) |  |  |
| OR (95%CI) | 1.37 (0.71–2.67) | 1.33 (0.67–2.63) |
| p-value | 0.347 | 0.412 |
| Penile length (≥8.0cm vs. <8.0cm) |  |  |
| OR (95%CI) | 1.10 (0.60–2.02) | 0.88 (0.46–1.67) |
| p-value | 0.758 | 0.691 |
| Penile circumference (≥8.0cm vs. <8.0cm) |  |  |
| OR (95%CI) | 1.69 (0.92–3.14) | 1.60 (0.84–3.04) |
| p-value | 0.090 | 0.152 |
| Testis size (≥20mL vs. <20mL) |  |  |
| OR (95%CI) | 1.61 (0.88–2.99) | 1.50 (0.78–2.88) |
| p-value | 0.124 | 0.227 |
|  | Urinary bother (≥85) | |
|  | Univariate analysis | Multivariate analysis |
| Age (≥70 years vs. <70 years) |  |  |
| OR (95%CI) | 0.48 (0.26–0.89) | 0.52 (0.27–0.99) |
| p-value | 0.020* | 0.049* |
| Medication for BPH (Positive vs. negative) |  |  |
| OR (95%CI) | 0.56 (0.28–1.09) | 0.64 (0.31–1.34) |
| p-value | 0.088 | 0.235 |
| Prostate volume (≥30 mL vs. <30 mL) |  |  |
| OR (95%CI) | 1.00 (0.54–1.85) | 1.13 (0.58–2.20) |
| p-value | 1.000 | 0.711 |
| Nerve sparing (Bilateral/Unilateral vs. non) |  |  |
| OR (95%CI) | 1.77 (0.91–3.55) | 1.79 (0.88–3.64) |
| p-value | 0.096 | 0.109 |
| Penile length (≥8.0cm vs. <8.0cm) |  |  |
| OR (95%CI) | 1.22 (0.66–2.26) | 1.05 (0.54–2.03) |
| p-value | 0.523 | 0.887 |
| Penile circumference (≥8.0cm vs. <8.0cm) |  |  |
| OR (95%CI) | 1.57 (0.85–2.92) | 1.41 (0.73–2.73) |
| p-value | 0.152 | 0.204 |
| Testis size (≥20mL vs. <20mL) |  |  |
| OR (95%CI) | 1.11 (0.60–2.04) | 0.97 (0.50–1.90) |
| p-value | 0.748 | 0.933 |
|  | Urinary irritation/obstruction (≥90) | |
|  | Univariate analysis | Multivariate analysis |
| Age (≥70 years vs. <70 years) |  |  |
| OR (95%CI) | 0.46 (0.25–0.86) | 0.50 (0.26–0.96) |
| p-value | 0.015* | 0.038* |
| Medication for BPH (Positive vs. negative) |  |  |
| OR (95%CI) | 0.67 (0.34–1.30) | 0.73 (0.35–1.55) |
| p-value | 0.235 | 0.414 |
| Prostate volume (≥30 mL vs. <30 mL) |  |  |
| OR (95%CI) | 1.05 (0.57–1.95) | 1.05 (0.53–2.06) |
| p-value | 0.875 | 0.895 |
| Nerve sparing (Bilateral/Unilateral vs. non) |  |  |
| OR (95%CI) | 1.68 (0.85–3.40) | 1.83 (0.88–3.82) |
| p-value | 0.133 | 0.106 |
| Penile length (≥8.0cm vs. <8.0cm) |  |  |
| OR (95%CI) | 1.66 (0.90–3.12) | 1.59 (0.81–3.12) |
| p-value | 0.107 | 0.178 |
| Penile circumference (≥8.0cm vs. <8.0cm) |  |  |
| OR (95%CI) | 1.77 (0.95–3.32) | 1.66 (0.84–3.26) |
| p-value | 0.073 | 0.142 |
| Testis size (≥20mL vs. <20mL) |  |  |
| OR (95%CI) | 0.80 (0.43–1.48) | 0.62 (0.31–1.25) |
| p-value | 0.469 | 0.183 |
|  | Urinary incontinence (≥75) | |
|  | Univariate analysis | Multivariate analysis |
| Age (≥70 years vs. <70 years) |  |  |
| OR (95%CI) | 1.07 (0.58–2.00) | 1.15 (0.60–2.21) |
| p-value | 0.823 | 0.672 |
| Medication for BPH (Positive vs. negative) |  |  |
| OR (95%CI) | 0.61 (0.30–1.21) | 0.58 (0.27–1.23) |
| p-value | 0.158 | 0.155 |
| Prostate volume (≥30 mL vs. <30 mL) |  |  |
| OR (95%CI) | 1.28 (0.69–2.40) | 1.51 (0.78–2.93) |
| p-value | 0.429 | 0.226 |
| Nerve sparing (Bilateral/Unilateral vs. non) |  |  |
| OR (95%CI) | 1.42 (0.72–2.80) | 1.40 (0.70–2.81) |
| p-value | 0.312 | 0.347 |
| Penile length (≥8.0cm vs. <8.0cm) |  |  |
| OR (95%CI) | 1.02 (0.55–1.90) | 0.82 (0.42–1.59) |
| p-value | 0.950 | 0.557 |
| Penile circumference (≥8.0cm vs. <8.0cm) |  |  |
| OR (95%CI) | 1.52 (0.82–2.85) | 1.50 (0.77–2.89) |
| p-value | 0.184 | 0.230 |
| Testis size (≥20mL vs. <20mL) |  |  |
| OR (95%CI) | 1.45 (0.78–2.71) | 1.29 (0.66–2.51) |
| p-value | 0.243 | 0.459 |

Expanded Prostate Cancer Index Composite: EPIC, OR: odds ratio, CI: confidence interval, BPH: benign prostate hyperplasia. *p < 0.05
