# Supplementary material for: Association Between Preoperative Penile Circumference and Urinary Function After Robot‐Assisted Radical Prostatectomy
Source: Int J Urol. 2025 Jul 18;32(11):1576–86. doi: 10.1111/iju.70179 (PMC12586765; doi:10.1111/iju.70179)
Supplement: Supplementary file 5 — Table S3. Univariate and multivariate logistic regression analyses for predictors of high total IPSS and IPSS‐QOL. BPH, benign prostate hyperplasia; CI, confidence interval; IPSS, International Prostate Symptom Score; OR, odds ratio; QOL, quality of life, *p < 0.05. [file IJU-32-1576-s004.docx]

Supplementary Table 3

Univariate and multivariate logistic regression analyses for predictors of high total IPSS and IPSS-QOL.

| Clinicopathological factors | Total IPSS (≥9) | |
| --- | --- | --- |
|  | Univariate analysis | Multivariate analysis |
| Age (≥70 years vs. <70 years) |  |  |
| OR (95%CI) | 1.50 (0.78–2.91) | 1.39 (0.70–2.78) |
| p-value | 0.228 | 0.345 |
| Medication for BPH (Positive vs. negative) |  |  |
| OR (95%CI) | 1.08 (0.51–2.23) | 1.02 (0.46–2.28) |
| p-value | 0.833 | 0.377 |
| Prostate volume (≥30 mL vs. <30 mL) |  |  |
| OR (95%CI) | 0.77 (0.42–1.41) | 0.89 (0.44–1.80) |
| p-value | 0.396 | 0.746 |
| Nerve sparing (Bilateral/Unilateral vs. non) |  |  |
| OR (95%CI) | 0.65 (0.75–3.23) | 0.60 (0.28–1.31) |
| p-value | 0.238 | 0.202 |
| Penile length (≥8.0cm vs. <8.0cm) |  |  |
| OR (95%CI) | 0.90 (0.47–1.74) | 0.99 (0.50–2.00) |
| p-value | 0.758 | 0.986 |
| Penile circumference (≥8.0cm vs. <8.0cm) |  |  |
| OR (95%CI) | 0.47 (0.24–0.92) | 0.48 (0.24–0.97) |
| p-value | 0.027* | 0.040* |
| Testis size (≥20mL vs. <20mL) |  |  |
| OR (95%CI) | 1.26 (0.65–2.44) | 1.40 (0.68–2.88) |
| p-value | 0.488 | 0.357 |
|  | IPSS QOL (≥4) |  |
|  | Univariate analysis | Multivariate analysis |
| Age (≥70 years vs. <70 years) |  |  |
| OR (95%CI) | 0.89 (0.46–1.69) | 0.77 (0.34–1.54) |
| p-value | 0.716 | 0.458 |
| Medication for BPH (Positive vs. negative) |  |  |
| OR (95%CI) | 1.66 (0.82–3.33) | 1.76 (0.81–3.83) |
| p-value | 0.156 | 0.152 |
| Prostate volume (≥30 mL vs. <30 mL) |  |  |
| OR (95%CI) | 0.72 (0.37–1.37) | 0.65 (0.32–1.30) |
| p-value | 0.315 | 0.221 |
| Nerve sparing (Bilateral/Unilateral vs. non) |  |  |
| OR (95%CI) | 0.61 (0.29–1.24) | 0.59 (0.28–1.28) |
| p-value | 0.175 | 0.185 |
| Penile length (≥8.0cm vs. <8.0cm) |  |  |
| OR (95%CI) | 0.61 (0.31–1.16) | 0.73 (0.37–1.46) |
| p-value | 0.133 | 0.378 |
| Penile circumference (≥8.0cm vs. <8.0cm) |  |  |
| OR (95%CI) | 0.54 (0.28–1.04) | 0.56 (0.28–1.12) |
| p-value | 0.067 | 0.100 |
| Testis size (≥20mL vs. <20mL) |  |  |
| OR (95%CI) | 0.85 (0.44–1.61) | 1.04 (0.51–2.11) |
| p-value | 0.610 | 0.919 |

IPSS: International Prostate Symptom Score, QOL: quality of life, OR: odds ratio, CI: confidence interval, BPH: benign prostate hyperplasia. *p < 0.05
